# Use of new technologies and promotion of breastfeeding: integrative literature review

**DOI:** 10.1590/1984-0462/2022/40/2020234

**Published:** 2021-09-01

**Authors:** Dulce Maria Pereira Garcia Galvão, Ernestina Maria Batoca Silva, Daniel Marques Silva

**Affiliations:** a Escola Superior de Enfermagem de Coimbra, Coimbra, Portugal.; b Escola Superior de Saúde de Viseu, Instituto Politécnico de Viseu, Viseu, Portugal.; c Escola Superior de Enfermagem de Coimbra, Unit of Health Sciences Investigation – Nursing, Coimbra, Portugal.

**Keywords:** Breastfeeding, Social networking, Social media, Health promotion, Health personnel, Aleitamento materno, Rede social, Mídias sociais, Promoção da saúde, Pessoal de saúde

## Abstract

**Objective:**

To identify the most used social networks and the most consumed contents by women seeking support and further understanding of breastfeeding/breast milk.

**Data source:**

An integrative literature review was performed using the Psychology & Behavioral Sciences Collection, MEDLINE Complete, CINAHL Complete, MedicLatina, Academic Search Complete and ERIC databases. The search was conducted in April, 2020. The inclusion criteria were: publications in Portuguese, English or Spanish with several keywords, such as “Breastfeeding”, “Social Networking”, “Social Media”, “Breastfeeding Promotion”, in the title and in the abstract, with the combination of the Boolean operators “AND” and “OR”, in original articles of primary source, which were available in full text and were published between 2015 and 2020.

**Data synthesis:**

Out of the 93 articles that were first examined, 10 were used in the descriptive summary. Studies from the United States, Sweden, New Zealand, Brazil, Australia, Indonesia, and Switzerland were included in the review. Women were found to use several social networks, which is facilitated by an easy access to the Internet and to its content through several electronic resources, often using more than one device simultaneously. Most issues were universally recognized as some of the most common reasons for interrupting breastfeeding.

**Conclusions:**

The analyzed studies show that women seek to clarify their doubts outside the traditional health services’ environment, using Facebook, apps, websites, online videos, podcasts and e-mail. We stress the importance of these support groups for promoting breastfeeding and the need for health professionals to introduce themselves in social networks to reach mothers.

## RESUMO

**Objetivo:**

Identificar as redes sociais mais utilizadas e os conteúdos mais consumidos pelas mulheres que procuram apoio e esclarecimento sobre amamentação/aleitamento materno.

**Fontes de dados:**

Revisão integrativa da literatura, nas bases de dados Psychology & Behavioral Sciences Collection, MEDLINE Complete, CINAHL Complete, MedicLatina, Academic Search Complete e Education Resources Information Center (ERIC). A busca foi realizada em abril de 2020. Os critérios de inclusão foram: publicações em português, inglês ou espanhol com vários descritores, tais como Breast-Feeding, Social Networking, Social Media, Breast-Feeding Promotion, no título e resumos, com a combinação dos operadores booleanos AND e OR, estudos originais de natureza primária, disponibilizados na íntegra e publicados entre 2015 e 2020.

**Síntese dos dados:**

Dos 93 artigos inicialmente avaliados, dez compuseram a síntese descritiva. Foram envolvidos estudos dos Estados Unidos, Suécia, Nova Zelândia, Brasil, Austrália, Indonésia e Suiça. Observou-se que as mulheres utilizam uma variedade de redes sociais, estimuladas pelo fácil acesso à internet e ao seu conteúdo, mediante os mais diversos meios eletrônicos e muitas vezes em mais de um equipamento simultaneamente. A maioria das questões eram temas universalmente reconhecidos como alguns dos motivos mais comuns para a interrupção do aleitamento materno.

**Conclusões:**

Os estudos analisados evidenciam que as mulheres procuram no Facebook, aplicativos, sites, vídeos on-line, podcasts e e-mail o esclarecimento das suas dúvidas em vez de o ambiente tradicional dos serviços de saúde. Realçamos a importância desses grupos de apoio na promoção da amamentação e a necessidade de os profissionais de saúde se introduzirem nas redes sociais para chegarem às mães.

## INTRODUCTION

The benefits of breastfeeding and breast milk both for the child and the mother are well known.^1^ ^,^ ^2^ Breastfeeding is the strongest isolated strategy for the prevention of infant mortality, promotion of physical and mental health of the child and the woman who breastfeeds.^3^ Exclusive breastfeeding is recommended for the first six months of life of the child, as well as its maintenance with complementary foods until the age of 2 years or more.^4^ However, worldwide, only 40% of the children younger than 6 months of age are exclusively breastfed, and three out of five newborns are not breastfed in the first hour of life.^4^ Other data show that only two out of five children younger than six months of age are exclusively breastfed.^2^ In Portugal, in 2014, the prevalence of exclusive breastfeeding at 3, 4 and 6 months of age was 55.9, 48.5, and 30.3%, respectively.^5^

Acknowledging breastfeeding as the best source of nutrition for newborns and children constitutes a global goal: that until 2025 at least 50% of the children be exclusively breastfed in the first six months of life.^6^ There are several factors to explain the early abandonment of breastfeeding. Raising awareness among the women is essential and should be one of the efforts in all countries.^7^ In fact, studies have shown that the implementation of pro-breastfeeding interventions in health systems and in the community has the potential to increase the rates of exclusive breastfeeding 2.5 times; however, mothers need to have access to information and support to breastfeed immediately after birth. There should also be rules to support and encourage breastfeeding, even in public spaces. In the communities, the support of advisors and trained colleagues, including other mothers and relatives, is equally relevant. Also, the support of men, husbands and partners cannot be underestimated.^7^

In order to promote, protect and support breastfeeding, several initiatives have been implemented around the world. We emphasize the initiative *Hospitais Amigos dos Bebês*, which establishes the adoption of ten measures for the success of breastfeeding, divided in two groups: clinical management procedures and key clinical practices.^4^ In these recommendations, the following steps stand out:

- To have a written policy of breastfeeding promotion that is regularly shared with the entire health care staff and the parents;
- To discuss the advantages and the practice of breastfeeding with all pregnant women;
- To help mothers to recognize the request for breastfeeding from their babies and react to them;
- To inform the mothers about the risks of using bottles, artificial nipples and pacifiers;
- To coordinate discharge from the hospital or maternity ward so that parents and infants can have access to the existing support and care services.

Even though this initiative determines the encouragement and on-site support to breastfeeding by health professionals,^8^ its motivation is essential for the decision to breastfeed, depending on the personal beliefs of the woman and the support she receives from her family and from society.^9^ In this sense, it is observed that women often do not dispose of these groups, both in the community and in the family, and end up being supported by a smaller network.^8^

To fulfill this gap, women started to look for support and orientation in the social media,^8^ in their computers, tablets and smartphones.^10^ In fact, social media became the favorite internet tool for consumers, and is considered as the most important mean of communication in this environment.^11^ It is a large online support network, stimulated by the easy access to the internet and its content through several electronic means. The available information has become more specialized; however, it is a new and little studied resource, and a promising field for the development of research.^8^

Therefore, we carried out this study whose objective is to identify the most used social media and the most consumed contents by women who look for support and clarification on the topics of breastfeeding/breast milk. It is important to mention the scenario of scientific evidence in the social media about breastfeeding, since this is an important mean to publish information.

## METHOD

We performed an integrative literature review, according to the classification of level of evidence and the six recommended steps:^12^ ^,^ ^13^ selection of the theme and guiding question; establishment of inclusion and exclusion criteria; sample (article selection); categorization of the selected articles; data analysis and interpretation; and synthesis of the knowledge through the presentation of an integrative review.

In the first step, the following guiding question was defined: What are the most used social media and most consumed contents by women who look for support and clarification on breastfeeding/breast milk?

**Figure 1:**
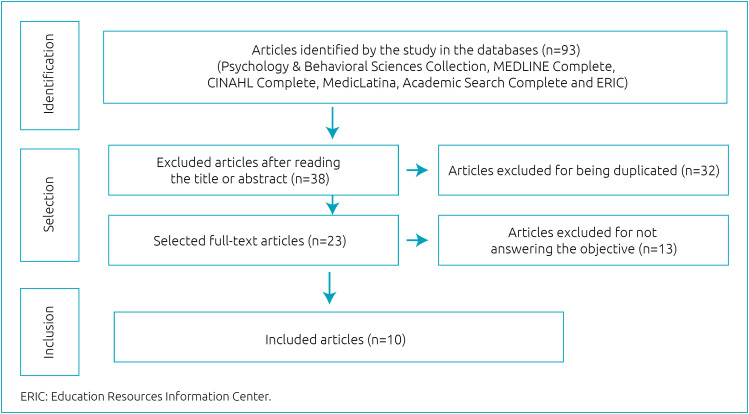
Flowchart of the stages of identification, selection and inclusion of articles.

In the second step, the inclusion criteria were the articles with samples of women who look for support and clarification about breastfeeding/breast milk in the social media, classified as primary source, in Portuguese, English or Spanish, with free access, available in full and published between 2015 and 2020. We excluded review articles. The article was revised by two independent researchers, in April, 2020, accessing the databases Psychology & Behavioral Sciences Collection, MEDLINE Complete, CINAHL Complete, MedicLatina, Academic Search Complete and Education Resources Information Center (ERIC). The research was carried out observing titles and abstracts, using the descriptors: Networking Social, Networking Social On-line, Facebook, Social Media, Twitter, LinkedIn, Instagram, E-technologies, Digital Technology, Electronic Technology, Mobile Devices, Internet, Technology and Breastfeeding, Lactation, Breastfeeding Promotion, Lactation Promotion, Human Milk Promotion, and the Boolean operators *AND* / *OR*. In general, we recovered 93 articles from the databases, and after identification and exclusion, by reading the title or abstract (38) and duplicates (32), 23 articles were evaluated with full texts. In this stage, we excluded 13 articles for not providing answers to the objective. Therefore, ten articles were included for data extraction (Figure 1).

## RESULTS

The analysis allowed access to ten studies, which were included in this review for providing an answer for the defined question and objective. Nine studies were published in English and one in Portuguese; two in 2016, two in 2017, three in 2018, and three in 2019, with great diversity regarding the location of the publication, in terms of continents and countries. As to the type of study, three are quantitative and seven are qualitative. One of them fits level of evidence II, and the others, level of evidence VI. Table 1 presents the summary of the characteristics of the included studies.

**Table 1:** Characteristics of the included articles (n=10).

Authors	Country/year of publication	Type of study	Level of evidence	Studied population/sample
Demirci et al.^14^	United States (2016)	Quantitative/randomized	II	146 puerperal mothers of NB who were born with 34 to 37 gestational weeks, with intention to breastfeed
Tomfohrde e Reinke^15^	United States (2016)	Quantitative/descriptive	VI	309 participants
Power et al.^16^	United States (2017)	Quantitative/descriptive/cross-sectional	VI	975 or 48.9% of the authorized representatives of Women, Infants, and Children (WIC) in the delta of River Yukon Kuskokwim (YKD), in Southwest Alaska, were randomly selected.
Wennberg et al.^17^	Sweden (2017)	Qualitative/descriptive/cross-sectional	VI	370 published posts in 2 online forums
Alianmoghaddam et al.^18^	New Zealand (2018)	Qualitative/descriptive	VI	30 puerperal women
Araújo et al.^19^	Brazil (2018)	Qualitative/descriptive: case report	VI	30 pregnant women who accompanied their pregnancy at a basic health unit in different gestational periods
Bridges et al.^20^	Australia (2018)	Qualitative/etnographic	VI	778 posts on Facebook
Dewanti et al.^21^	Indonesia (2019)	Qualitative/descriptive	VI	10 mothers who were breastfeeding
Rezaallah et al.^22^	Switzerland (2019)	Qualitative/descriptive	VI	*Posts* in 21 pregnancy forums that were publically available
Wagg, Callanan & Hasset.^23^	United States (2019)	Qualitative/descriptive	VI	501 posts collected between November 1st and 7, 2016, in an online breastfeeding support group

NB: newborn.

After reading the articles, we grouped the most relevant information of each article (Table 2). Therefore, we created four blocks: used social network/information technology, consumed themes/contents, advantages of social media, and problems or difficulties with the use of social media.

**Table 2:** Synthesis of the information available by the included articles.

Available information	
**Social networks/information technologies**	
	Apps and websites about pregnancy/parenthood and breastfeeding support.^14^ ^,^ ^18^ ^,^ ^19^ ^,^ ^21^
	E-mail.^14^ ^–^ ^16^
	Internet.^14^ ^,^ ^16^ ^,^ ^18^ ^,^
	Online vídeos.^14^ ^,^ ^16^
	Online courses/classes, electronic medical journals, peer forums and e-books.^14^
	Podcasts.^14^ ^,^ ^17^ ^,^ ^20^ ^,^ ^22^ ^,^ ^23^
	Text messages.^14^ ^,^ ^16^
	Facebook.^14^ ^–^ ^16^ ^,^ ^18^ ^,^ ^20^
	Google.^18^
	Pinterest, Twitter, Instagram, BabyCenter and Glide.^15^
	Smartphones, tablets and computers.^16^ ^,^ ^18^
	Skype or telephone.^18^
	WhatsApp.^19^
	Online forums.^22^
**Consumed themes/contents**	
	Support and encouragement to breastfeeding, besides its promotion.^14^ ^,^ ^18^ ^,^ ^20^
	Empowerment and confidence in parental skills: advantages and techniques of breastfeeding *versus* problems with breastfeeding.^14^ ^,^ ^17^ ^,^ ^20^
	Concerns about the early introduction of formula.^17^ ^,^ ^23^
	Imformation about breastfeeding and child nutrition.^14^ ^,^ ^18^ ^,^ ^19^
	Information about nutrition,^20^ ^,^ ^23^ sexual activity, physical activities, medications,^23^ oral health, vaccines, tests, body and emotional changes during pregnancy.^19^
	Information about types of delivery, importance of family support, newborn vaccination, mother and child care network.^19^
	Information about breastfeeding and work, including the collection and storage of breast milk.^20^ ^,^ ^23^
	Information about maternal health.^21^ ^,^ ^23^
	Information about child care and health.^21^ ^,^ ^23^
	Information about pregnancy, breastfeeding and use of medication for multiple sclerosis.^22^
**Advantages of social networks**	
	To provide support and education outside the traditional doctor’s office^14^ ^,^ ^19^ or to people who live in distant communities.^16^
	Health professionals promoting the practice or publicizing scientific and clinically solid information about breastfeeding and other aspects of maternity.^15^
	To develop better support for mothers, helping them feel more confident in their parental skills, from health professionals.^17^
	To facilitate the dissemination of information about breastfeeding and child nutrition.^18^
	To share experiences about pregnancy,^19^ breastfeeding and child care.^21^ ^,^ ^23^
	To be an easy way of accessing information, of emotional help and encouragement.^21^
	To socialize with other mothers.^21^
	To clarify doubts with health professionals involved in pregnancy or child care.^22^
**Problems or difficulties to use the social media**	
	The use of technologies while breastfeeding can lead mothers to miss the opportunity to make eye contact and interact with their children.^15^
	The non-use of technology is owed to not having access to the internet/computer and the high cost of internet.^16^
	Overwhelming amount of information and taking up too much time.^21^

### Used social media / information technology

The study carried out by Demirci et al.^14^ about the use and preferences regarding technology to obtain perinatal and breastfeeding support observed that the most used technologies were applications, internet, websites about pregnancy/parenthood and e-mail. Other additional technological sources were used, as follows: Facebook, text messages, videos and online courses/classes, electronic medical journals, peer forums, e-books and podcasts.^14^

Besides, Tomfohrde and Reinke,^15^ in the study carried out to collect information about breastfeeding and social media, observed that of the participants who indicated using social media or e-mail while breastfeeding, 92% reported using Facebook. The participants pointed out to the use of other social media platforms: Pinterest, Twitter, Instagram, BabyCenter and Glide.

Likewise, Power et al.^16^ verified, in the study developed to analyze the use of media technology in native communities of Alaska about nutrition education, that the use of media technology was common. The interviewees reported having access to a wide variety of technologies, such as smartphones (78.8%), tablets (44.8%) and computers (38.4%). Text messages were the most used one, followed by Facebook. In total, 80.3% of the interviewees used the internet. The most popular ways to receive information about nutrition were e-mail (67.8%), online videos (60.4%), Facebook (58.0%) and text messages (54.4%).

Likewise, Wennberg et al.,^17^ who were based on the analysis of 370 published posts, and Alianmoghaddam et al.,^18^ who explored the influence of social media on the practice of exclusive breastfeeding, observed that most mothers (22 out of 30) used the internet and social media to support their breastfeeding practice. The most used research support means and mechanisms were Google, Facebook and websites addressed to parents. They equally verified that some of the participants, whose relatives lived in other countries, used Skype or the telephone. On the other hand, the participants in the studies carried out by Wennberg et al.,^17^ Araújo et al.,^19^ Bridges et al.,^20^ Dewanti et al.,^21^ Rezaallah et al.^22^ and Wagg et al.^23^ observed posts that were published in online forums, the use of WhatsApp as an education tool and health promotion among pregnant women, in prenatal care, closed Facebook groups about breastfeeding support and online groups that support mothers.

### Consumed themes/contents

Support, encouragement and expectations about the evolution of breastfeeding in the postpartum period, signs of adequate production of milk and child nutrition, visual representations of how to properly position the child and advice from experts and colleagues were the most consumed contents by puerperal women in the study by Demirci et al.^14^ Besides, of the interviews with the participants of the study carried out by Alianmoghaddam et al.,^18^ four themes came up:

- The Y generation mothers need online information about child nutrition;
- Apps for smartphones can be a good option to promote breastfeeding;
- Weak bonds between mothers who breastfeed, according to Facebook and other social media websites, facilitate the dissemination of information;
- The use of child nutrition support from a geographical distance, via Skype.

Likewise, of the 72 specific questions about breastfeeding, Bridges et al.^20^ categorized 55 (76%) in three areas: breastfeeding management; breastfeeding and health; and breastfeeding and work. “Balance between social expectations and confidence in your parental skills”, “Making an effort to be a good mother”, “Making an effort for your own well-being”, and “Making an effort to find your own path” are emphasized by Wenberg et al..^17^ On the other hand, Araújo et al.,^19^ Dewanti et al.,^21^ Rezaallah et al.^22^ and Wagg et al.^23^ stated that the study participants showed doubt regarding the changes in the different gestational periods, diet, sexual activity during pregnancy, self-medication, oral health, vaccines during pregnancy, tests, body and emotional changes, child care, breastfeeding, types of delivery, importance of family support, physical activities, vaccination of the child and maternal and child care network.

### Advantages of social media

The results of the different studies showed that the omnipresent nature of technology represents an opportunity to provide support and education to women who would not receive it in another way, for example, in the traditional doctor’s office.^14^ ^,^ ^19^ It allows the support of child nutrition in geographically distant populations^16^ and informs pregnant women not only about pregnancy itself, but also about delivery, the postpartum period, breastfeeding, child nutrition^18^ and child care.^21^ ^,^ ^23^ It is a mean that enables to share fears, concerns and exchange experiences between pregnant women of different ages and gestational periods^19^ to get emotional help, encouragement, besides being a way to socialize with other mothers.^21^

### Problems or difficulties to use social media

According to the study by Tomfohrde and Reinke,^15^ the use of technology while breastfeeding can lead mothers to miss the opportunity to make eye contact and interact with their children. Besides, Dewanti et al.^21^ emphasized that some mothers manifested that, due to the use of social media, they felt overwhelmed by the amount of information and because it takes up too much time.

Power et al.^16^ observed that, of the 63 interviewees who did not use the internet, the most common barriers for its use were not having access to it (36.4%), not having access to a computer (28.8%) and due to its high cost (13.6%). Only two (3%) interviewees who did not use the internet reported that they were simply not interested in it. The possible barriers to receiving nutrition information through media technology included slow internet (50.1%), no access to computers (41.7%) and high cost of the internet (34.9%).

## DISCUSSION

The use of new information and communication technology has increased in the past years,^24^ and the current generation is known as the Y generation, or the “internet generation”.^18^ Women at reproductive age, pregnant women or mothers of one child use new technologies and look, in the social media, for information about pregnancy, birth and child care.^8^ ^,^ ^14^ ^–^ ^16^ ^,^ ^18^ ^,^ ^19^ ^,^ ^21^ The social network is a low-cost strategy in the improvement and in the health care of the mother and the child.^8^

In fact, nowadays the mothers have a wide online support network, stimulated by the easy access to the internet and its content through several electronic means, often in more than one device at a time: computers, notebooks, smartphones, tablets and cell phones,^8^ ^,^ ^10^ ^,^ ^14^ ^–^ ^16^ ^,^ ^18^ which allows them to be part of communities, groups, pages, blogs and social networks (Facebook, apps and websites, online videos, podcasts and e-mail), sharing knowledge and experiences with other people in similar situations.^10^ ^,^ ^14^ ^–^ ^16^ ^,^ ^18^ ^–^ ^23^ However, some mothers report unfavorable experiences for feeling overwhelmed by too much information, then diverting the attention from the main goals; besides, it takes up too much of their time.^21^

The most consumed themes/contents in the internet by the mothers are information about themselves or their children, and especially about breastfeeding,^9^ ^,^ ^25^ pregnancy, delivery, returning to work, food introduction and raising children.^8^ Similar data were observed in the studies of our review,^14^ ^,^ ^17^ ^–^ ^23^ which showed that the most researched topics were related to pregnancy progress and fetal development, general questions and concerns about pregnancy, use of medications during the reproductive period, labor, delivery, postpartum period and child care, breastfeeding, breastfeeding and health, breastfeeding and work, breastfeeding and medication, milk collection and storage, infant health care, introduction of solid foods and replacements for breast milk.

It is common that women who breastfeed, especially those breastfeeding for the first time, have doubts about their abilities and the way to breastfeed their children. There are many situations related both to the mother and the child that can be in the base of early abandonment of breastfeeding. In the analyzed studies,^14^ ^,^ ^17^ ^,^ ^20^ the authors verified that most questions regarded themes that are recognized as some of the main reasons to interrupt breastfeeding (time and frequency, breast refusal, position and placement of the nipple, mastitis, maternal diseases and breastfeeding, human milk storage, dealing with the difficulties of breastfeeding, providing and producing milk).

It is important to mention that women look to clarify their doubts in the social network, and not in the traditional medical services. In this sense, this is a warning for health professionals to participate in these spaces and reach the mothers^8^, thus providing clarifications about the adequate techniques and the previous preparation for the difficulties that may come with breastfeeding.^26^ Knowing the social media addressed to the women is very important, so that one can identify the most influential individuals and understand the interaction of these people with the women in the breastfeeding process.^27^ A suggestion for future studies is the development of analyses that evaluate the interventions carried out by health professionals in online social support groups to support breastfeeding.^23^

Finally, as a limitation of this review, we mention that the results reveal the reality of specific sociocultural contexts in the countries where these studies were conducted. Besides, the fact that the studies were mostly qualitative and had a great variety of contents published in the social media made it difficult to conduct a more comparable analysis.
